# Behavioral flexibility promotes collective consistency in a social insect

**DOI:** 10.1038/s41598-018-33917-7

**Published:** 2018-10-26

**Authors:** Linda Karen Garrison, Christoph Johannes Kleineidam, Anja Weidenmüller

**Affiliations:** Department of Biology, University of Konstanz, Universitätsstrasse 10, 78457 Konstanz, Germany

## Abstract

Deciphering the mechanisms that integrate individuals and their behavior into a functional unit is crucial for our understanding of collective behaviors. We here present empirical evidence for the impressive strength of social processes in this integration. We investigated collective temperature homeostasis in bumblebee (*Bombus terrestris*) colonies and found that bees are less likely to engage in thermoregulatory fanning and do so with less time investment when confronted with heat stress in a group setting than when facing the same challenge alone and that this down-regulation of individual stimulus-response behavior resulted in a consistent proportion of workers in a group engaged in the task of fanning. Furthermore, the bees that comprised the subset of fanning individuals changed from trial to trial and participation in the task was predominately unpredictable based on previous response behavior. Our results challenge basic assumptions in the most commonly used class of models for task allocation and contrast numerous collective behavior studies that emphasize the importance of fixed inter-individual variation for the functioning of animal groups. We demonstrate that bumblebee colonies maintain within-group behavioral heterogeneity and a consistent collective response pattern based on social responsiveness and behavioral flexibility at the individual level.

## Introduction

Group-living animals frequently coordinate their behavior when facing challenges such as finding food and shelter or detecting and avoiding predators^1,2^. These collective behaviors are often self-organized – that is, group members locally influence each other, and the modulations of individual actions result in a coordinated group-level outcome^3,4^. Thus, to understand how collective behaviors arise, we must unravel the modulatory processes that integrate individuals and their behavior into a functional collective unit.

The processes involved in this integration are bound to vary depending on the life history of a species, the selection pressures that the collective faces, and on the specifics of the challenge at hand. For instance, collective behaviors that involve the response of all group members (e.g. coordinated movement^5^) require different mechanisms of modulation and integration than collective behaviors based on unevenly distributed or heterogeneous contribution of groups members (e.g. shared vigilance^6^ or social cognition^7^). To the best of our knowledge, the extent to which these differences impact the within-group mechanisms of behavioral modulation has not been explicitly considered.

As individuals in collectives inherently behave in a social context, social feedback processes often play an important role in shaping individual behavior. Despite evidence that behavioral responses are influenced by the social context in which they occur^8^, previous studies often lack a precise characterization of how each individual responds to its environment and how this response is modulated by the social context (but see^9^). Such characterization requires the ability to modify the social context while maintaining an otherwise consistent sensory environment, which is experimentally challenging. Consequently, we still have a limited understanding of the modulation of individual behavior within a group and how this modulation, in turn, shapes collective properties.

Social insects are excellent models for such explorations; they present an amazing array of complex collective behaviors underlain by tens to millions of individuals performing numerous tasks in parallel^10–13^ and thus offer the opportunity to analyze the modulatory effect of social context on individual behavior in a system based on unevenly distributed responses among group members. Here, we studied collective temperature homeostasis in bumblebees (*Bombus terrestris*) to investigate if and how individual behavior is modulated by social context, and how this modulation contributes to a collective outcome. To ensure rapid brood development and colony growth^14,15^, colonies collectively maintain high and stable nest temperature. Underlying the collective temperature homeostasis is a subset of workers directly incubating the brood when temperatures are low and ventilating the brood by fanning their wings when temperatures are high^16,17^. As the thermoregulatory actions of individuals have a local effect, and individuals show heterogeneity in their thermoregulative responses^18–22^, homeostasis depends on a collective response.

**Figure 1:**
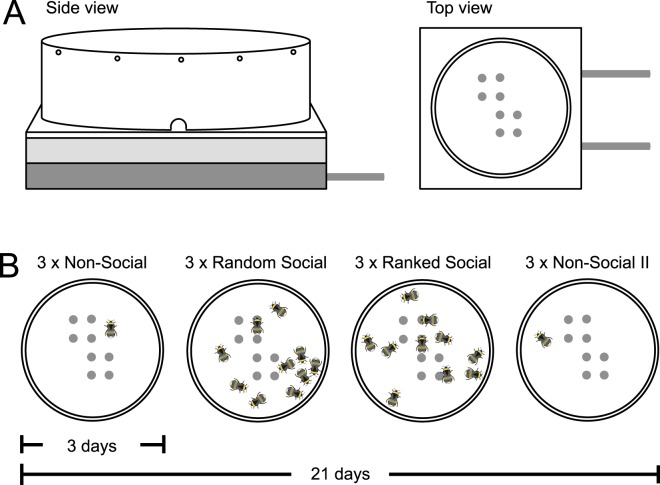
Experimental procedure. (**A**) Eight temperature-controlled brood dummies protruded from a metal heating plate through an insulation layer and the arena floor into each test arena. (**B**) Individuals were exposed to an increase in brood dummy temperature once a day and tested three times each on three consecutive days in four different contexts: alone (non-social context), in randomly assembled groups of ten (random social context) with each of the three trials consisting of the same 10 individuals, in low heterogeneity groups ranked according to their fanning behavior shown previously in the random social context (ranked social context), and alone again (non-social context II).

We assessed the fanning behavior of bumblebee workers when faced with an increase in brood temperature both alone and with nest mates present, as well as the resulting collective response pattern. To explicitly disentangle an individual’s response to the brood temperature stimulus from the influence of other group members, we used experimental arenas with a tightly controlled temperature environment (Fig. 1A) and manipulated the social environment while maintaining stimulus intensity and ensuring task availability. We tested 159 workers from 14 colonies in three different social contexts, exposing each individual to an increase in brood temperature three times in each context (Fig. 1B), and analyzed three ecologically-relevant parameters of individual behavior: (1) *fanning response probability*, the number of trials in which the individual shows a fanning response; (2) *fanning response threshold*, the temperature at which an individual starts fanning; and (3) *fanning response duration*, the number of minutes an individual shows fanning behavior.

## Results

### Individual behavior, non-social vs. social

**Figure 2:**
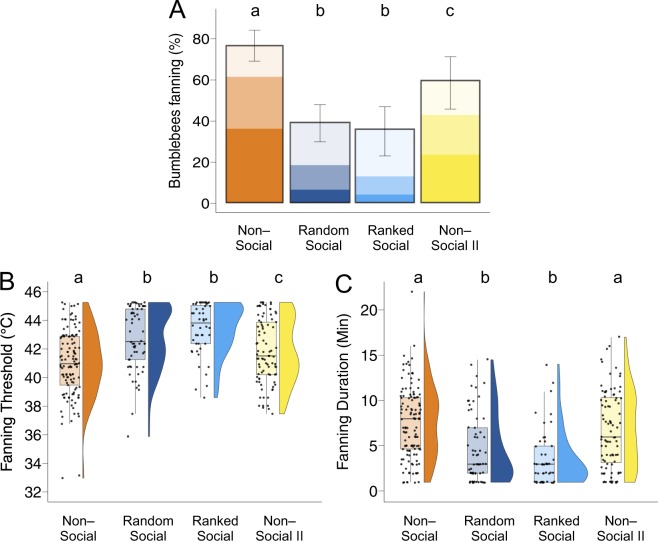
Fanning response parameters by context. (**A**) Percentage of bees tested (n = 159,158,78,77) that showed fanning. Transparency blocks represent percentage of bees that showed fanning once (lightest block), twice, or three times (darkest block) across the three trials of each context. Error bars show 95% confidence interval based on generalized liner mixed model (GLMM) fit to the data. (**B**) Boxplots and density plots depict mean fanning thresholds and (**C**) mean fanning durations. Each point depicts one individual (n = 121, 62, 48, 87). Sample sizes for A differ from B and C; in Rep. 1, 20 non-fanners in the non-social context and the random social context were not further tested in the ranked social context and the non-social context II, whereas all bees were tested across all four contexts in Rep 2.

**Figure 3:**
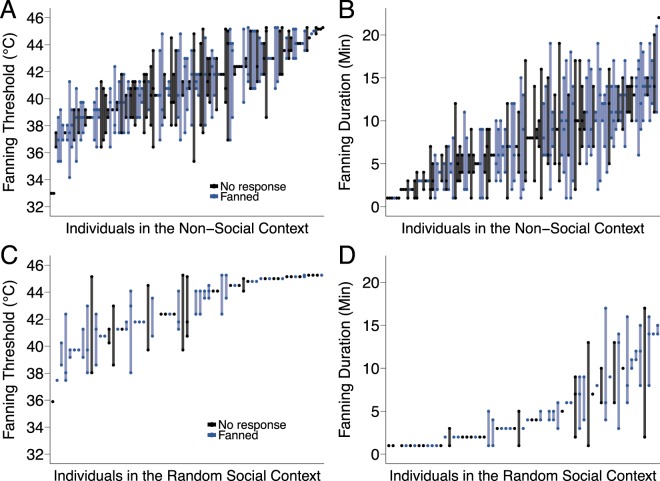
Inter-individual differences in fanning response. (**A**) Fanning thresholds and (**B**) fanning duration of each individual across three trials of the non-social context (n = 121). (**C**) Fanning thresholds and (**D**) fanning duration of each individual across three trials of the random social context  (n = 57). Individuals sorted by mean. (**A** and **B**) Blue denotes individuals that also fanned in the random social context. (**C** and **D**) Blue denotes individuals that also fanned in the ranked social context.

We first determined the response behavior of individuals when facing the brood temperature stimulus alone (non-social context; Fig. 1B). Of the 159 bees tested, 121 (77% [95% confidence interval: 68–84%]) engaged in the task of fanning at least once across three trials (Fig. 2A). Response probability per trial was 0.62 [0.52–0.70]. Mean fanning threshold was 40.87 °C [40.54–41.20] (Fig. 2B). We found inter-individual variation in fanning threshold (F = 1.837 p = 1.93e-4; repeatability RM = 0.231 [0.084–0.367], p = 0.00226; Fig. 3A). Mean fanning duration was 7.94 [7.30–8.57] minutes (Fig. 2C). We found inter-individual variation in fanning duration (F = 1.423 p = 0.0197; RM = 0.146 [0.00529–0.289], p = 0.0253; Fig. 3B). Individual threshold and duration were strongly correlated (R_119_ = 0.71, p < 2.2e-16); workers with low response thresholds tended to fan longer than workers with high response thresholds.

To test if social context provided by the presence of nest mates modulates individual stimulus-response behavior, we randomly allocated individuals to groups of 10 bees (n = 16 groups) and determined their response behavior in a group setting (random social context; Fig. 1B). All parameters of individual response behavior changed (Fig. 2; Table 1). Of the 158 bees tested, 62 (39% [30–48%]) engaged with the task of fanning at least once (Z = 6.52, p < 0.0001; Fig. 2A). Response probability per trial decreased to 0.12 [0.08–0.18] (Z = 12.17, p < 0.0001). Mean fanning threshold increased to 42.67 °C [42.16–43.17] (T = −6.299, p < 0.0001; Fig. 2B) and mean fanning duration decreased to 4.94 [3.98–5.90] minutes (T = 5.687, p < 0.0001; Fig. 2C). For the 57 bees that responded in both the non-social and random social context, this pattern is the same (Fig. S2; Table S1): response probability decreased (Z = 5.334, p < 0.0001), threshold increased (T = −5.975, p < 0.0001), and duration decreased (T = 5.665, p < 0.0001). In summary, individual investment in the task decreased in a social context.

**Table 1:** Fanning response parameters by treatment and pairwise comparisons between treatments.

	Proportion (95% CI)	Probability (95% CI)	Threshold (95% CI)	Duration (95% CI)
Non-social	0.77 (0.68–0.84)	0.62 (0.52–0.70)	40.87 (40.54–41.20)	7.94 (7.30–8.57)
Random social	0.39 (0.30–0.48)	0.12 (0.08–0.18)	42.67 (42.16–43.17)	4.94 (3.98–5.90)
Ranked social	0.34 (0.23–0.47)	0.10 (0.06–0.16)	43.27 (42.64–43.89)	3.61 (2.44–4.79)
Non-social II	0.59 (0.45–0.71)	0.39 (0.29–0.51)	41.73 (41.33–42.14)	7.22 (6.45–7.99)
	**Proportion z-ratio, p-value**	**Probability z-ratio, p-value**	**Threshold t-ratio, p-value**	**Duration t-ratio, p-value**
Non-social – Random social	6.519, <0.0001	12.169, <0.0001	−6.299, <0.0001	5.687, <0.0001
Non-social – Ranked Social	5.740, <0.0001	10.335, <0.0001	−7.014, <0.0001	6.851, <0.0001
Non-social – Non-social II	2.604, 0.0455	4.272, 0.0001	−3.625, 0.0018	1.631, 0.3620
Random social – Ranked social	0.710, 0.8933	0.854, 0.8282	−1.543, 0.4122	1.855, 0.2487
Random social – Non-social II	−2.710, 0.0340	−6.610, <0.0001	3.055, 0.0126	−4.048, 0.0003
Ranked social – Non-social II	−3.070, 0.0115	−6.604, <0.0001	4.289, 0.0001	−5.476, <0.0001

Can we predict which individuals will perform in a social setting from their stimulus-response behavior in the non-social context? Based on the assumptions of response threshold models^23,24^, we predicted that low threshold individuals would respond earlier in groups than high response threshold individuals, thereby potentially removing the higher threshold individuals from the task. This was not the case in our experiment; response threshold in the non-social context did not predict if an individual engaged in fanning during the random social context (Z = 0.979, p = 0.3277, R^2^ = 0.00824; Fig. 3A). The interaction between response probability and fanning duration in the non-social context predicted whether or not an individual responded in the random social context, but with low predictive value (Z = 2.604, p = 0.00921, R^2^ = 0.0646). Mean fanning duration across the three trials of non-social as an independent factor also weakly predicted response in the random social context (Z = −2.096, p = 0.0361, R^2^ = 0.0412; Fig. 3B), and response probability alone even less so (Z = −1.433, p = 0.15196; R^2^ = 0.0163).

### Collective pattern, random social groups

**Figure 4:**
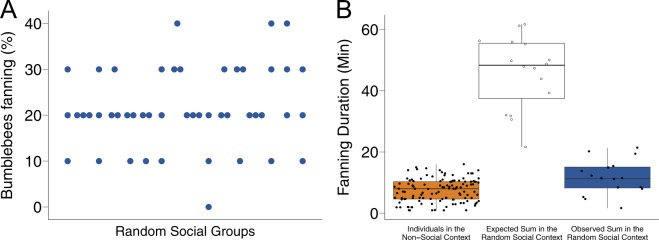
(**A**) Group-level fanning response, random social context. Percentage of bees in each group of ten bees (n = 16) that showed fanning behavior. Each group tested three times. 22% [18–26%] of workers per group engaged with the task of fanning. (**B**) Time invested into fanning, per test arena, observed vs. expected. (Left) Mean number of minutes each individual spent fanning when facing an increase in brood temperature alone (non-social context; n = 121). (Middle) Expected summed time spent fanning per group in random groups of ten individuals facing the same stimulus increase, if individuals had invested the same amount of time into fanning when in a group as when tested alone (n = 16). (Right) Observed summed time spent fanning per group and arena in groups of ten (random social context; n = 16).

How does this decrease in individual investment shape the collective response? At the group-level, 22% [18–26%] of the workers of a group in the random social context engaged with the task of fanning (Figs 4A, S3). If individual behavior had remained consistent from the non-social context to the random social context, we would have expected a fanning response in 58% [54–62%] of the workers in these groups (Z = 11.076, p < 0.0001). Social context mediates a down-regulation of individual responsiveness to an increase in brood temperature, resulting in a consistent group-level response pattern.

Colonies may be regulating not only the proportion of workers allocated to certain tasks but also collective time investment (Fig. 4B). If individuals had invested the same amount of time into dealing with the stimulus when in groups as when facing it alone, we would have found a total of 45.85 [41.94–49.76] minutes of fanning invested per test arena in groups of ten (Fig. 4B). However, the time collectively invested into fanning by all 10 workers of a group, 11.75 [7.84–15.66] minutes, is only slightly higher than the time invested on average by single individuals in the non-social context (T = −2.755, p = 0.0062; Fig. 4B) and lower than the expected summed duration of the 10 workers present (T = 12.446, p < 0.0001; Fig. 4B).

### Individual behavior, random social groups vs. ranked social groups

In order to test if the behavioral composition of a group further modulates individual stimulus-response behavior, we created groups with low heterogeneity in fanning behavior (ranked social context; Fig. 1B). We ranked workers according to their summed fanning duration across three trials of the random social context and assembled groups such that workers that had shown strong fanning in the random social context (“social fanners”) were now tested together and workers that had never shown fanning in the random social context were also tested together (Fig. S4).

There was no change in fanning response parameters from the random social context to the ranked social context (Fig. 2; Table 1). Of the 78 bees tested, 28 (34% [23–47%]) engaged in fanning at least once across three trials, response probability per trial was 0.10 [0.06–0.16], fanning threshold was 43.27 °C [42.64–44.89] and fanning duration was 3.61 [2.43–4.79] minutes (T = 1.855, p = 0.2487). Whether or not an individual responded when the social composition of a group changed could not be predicted based on her response shown in the random social context (response probability: Z = 1.428, p = 0.153; threshold: Z = −1.652, p = 0.0985; duration: Z = 1.753, p = 0.0796; Fig. 3C,D).

### Collective pattern, ranked social groups

**Figure 5:**
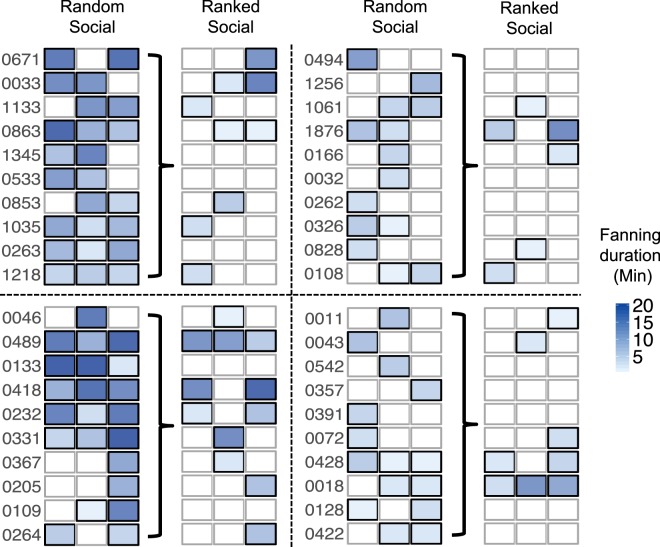
Fanning response of 40 individuals tested in the four ‘social fanner’ groups, in the ranked social context. Fanning response of individuals (indicated by their numbers) across three trials (columns) of the random social context and of the ranked social context. Gradient indicates fanning duration per trial; open boxes indicate no fanning. Individuals were assembled into groups of ten based on similarity in mean fanning duration shown in the random social context for testing in the ranked social context.

The unpredictability of individual contribution is further demonstrated by the group-level results of the four groups consisting of individuals which all showed fanning behavior in the random social context (Fig. 5). If individual behavior had remained consistent from the random social context, we would have found a 63% [54–71%] fanning response in these groups (Z = 5.083, p < 0.0001). However, only 30% [22–38%] of the workers in a group engaged in fanning per trial. Further, across the three trials, it was not the same subset of individuals contributing to the task (Fig. 5). Mean fanning duration per group was 15.75 [2.92–28.58] minutes, compared to the expected mean of 43.92 [31.08–56.75] minutes (T = 3.170, p = 0.0095) based on individual fanning duration in the random social context. We found little up-regulation of individual fanning responses in groups that consisted only of previous non-fanners (Fig. S4).

### Non-social II

To control for changes due to age, experience, and manipulation, individuals were again tested in a non-social context (non-social II; Fig. 1B). Of the 77 bees tested, 46 (59% [45–71%]) engaged in the fanning task at least once across the three final trials, demonstrating a return to higher individual task engagement from both social contexts (Fig. 2; Table 1). Response probability also increased to 0.62 [0.52–0.70], as did response threshold (40.87 [40.52–41.22]) and fanning duration (7.95 [7.23–8.68] minutes). Compared to the initial non-social context, fanning duration did not differ (T = 1.653, p = 0.3497), but the other parameters did (Fig. 2; Table 1). For the 32 individuals that responded in all four settings, there is no difference in response in the first non-social context to the second (Fig. S5; Table S2).

### Stimulus intensity & task availability

The temperature ramp enforced at the brood dummies minimized any cooling effect of fanning. Average brood dummy temperatures did not differ in any biologically relevant way between individual and group contexts (maximum difference between averaged dummy temperatures across ten trials in each context: 0.132 °C; Fig. S1). Task availability remained high in the group context (average dummy availability: 5.11 [5.03–5.18]; Fig. S1). Arena air temperature was slightly higher in the groups (maximum average difference between average air temperature: 1.10 °C; Fig. S1).

## Discussion

Our results demonstrate the modulatory strength of social context on the expression of individual behavior within collective thermoregulation in bumblebees. When exposed to an increase in brood dummy temperature without any nest mates present, a majority of bees showed fanning behavior. When these individuals were assembled into groups and exposed to the same stimulus, their probability to fan when facing that same stimulus challenge was strongly dampened. Further, bees that did respond in the social context generally showed higher response thresholds and shorter fanning durations compared to when tested alone, despite the consistent stimulus conditions between contexts (the task-relevant stimulus did not differ between the non-social and the social contexts, and task availability remained high throughout with no other tasks available). This demonstrates that individuals integrate personal information and social information (the presence and contributions of social group members) into their response decision and adjust their stimulus-response behavior when in groups.

Our findings are in contrast to a study on the effect of social context on fanning behavior in honeybees in which bees showed a higher fanning probability and lower fanning thresholds when in a group of three or ten compared to when tested alone^25^. The discrepancies between our results and those reported by Cook & Breed may be due to species specific or methodological differences. While bees in our study were fanning in response to overheating of the brood and were not exposed to heat stress themselves, bees in Cook & Breed were heated in glass jars without any brood or colony stimuli present. Fanning in this context may serve primarily aggregation purposes or the cooling of own body temperature and not occur in the context of the collective maintenance of brood temperature.

The dampening of individual fanning response found in our study resulted in a consistent collective response pattern. Groups exhibited strong behavioral heterogeneity with a large proportion of workers not fanning and a smaller subset of around 20–40% of the workers of a group taking on the task of fanning. If individuals had shown the same response as in isolation, around 60% of the workers of a group would have fanned. This pattern of decreased individual investment persisted even in groups composed exclusively of individuals that previously showed fanning in a group setting. Groups also showed a much lower total time investment compared to the expected summed fanning duration of the 10 workers present (around 10 minutes of fanning per group vs. expected 45 minutes). This pattern was persistent even though the stimulus, brood dummy temperature, could not be effectively down-regulated by the group, suggesting a strong social effect that is not indirectly mediated indirectly via an influence on the stimulus itself. In a study on fanning under heat stress in *Bombus terrestris* colonies, Weidenmüller^17^ found that colonies ranging in size from 10–119 workers consistently allocated no more than 33% of workers to the task of thermoregulation. As the same proportion are active in whole colonies as we found in our groups, this percentage of workers participating in the task of fanning and the amount of fanning time collectively invested seems to be sufficient to face a heat challenge and cool the brood in the nest under natural conditions.

Both positive and negative feedback play important roles in collective behavior^2,26^. We found negative social feedback integrating individuals into a collective homeostatic response. While studies on the effects of social context on individual and collective behavior often emphasize positive social feedback on individual behavior – namely amplification of behavioral response, behavioral conformity, or synchronization^2^, these have been reported predominately in collective behaviors that involve a behavioral response of all group members (e.g. collective movement^5^). Social context may have a very different effect on individual behavior during collective responses based on unevenly distributed responses among group members (e.g. shared vigilance^6^ or social cognition^7^). For such systems to function, only a subset of individuals needs to respond to a task-related stimulus. During shared vigilance in meerkats for example, only a few group members act as sentinels, even though the risk of predation is prevalent^27^. In cases of social cognition, there is always information in the environment to be learned, but only a subset of the population needs to have information for the group to perform well^28^. Similarly, in this study, there was always hot brood in need of cooling, but only a subset of bees performed the behavior. In these cases, negative social feedback mediating the down-regulation of individual stimulus-response behavior in the social context may be necessary to prevent overreaction of the system to a challenge^29^ and to ‘free up’ individuals for other tasks^30^.

What are the proximate mechanisms mediating the down-regulation of individual responsiveness and underlying the consistent group-level response? Here, we can only speculate. By performing a task, an individual will often modulate the task-related stimuli, which in turn will affect the behavior of its group members, reducing or increasing the likelihood of their engagement. We experimentally ruled out this feedback process; bees were confronted with the same increase in temperature across all contexts, and fanning did not change the stimulus. The fact that we still find a down-regulation in individual responsiveness demonstrates that social context directly affects the perception and/or evaluation of the task-relevant stimulus. The details of the social processes involved remain ambiguous. They could range from cues about the presence, activity or behavior of other bees, perceived via olfactory^31^ or vibrational cues to direct interactions^32–34^ and communication signals. It also remains to be shown if individual response behavior is down-regulated physiologically at the sensory periphery or centrally and if individuals are generally in a different motivational or responsive state when in a social group. We hypothesize that bumblebees make an assessment of the number of bees surrounding them and performing the task of fanning. Future studies analyzing individual movement patterns and interaction rates both with the brood dummy and with group members will provide insight into the details of these feedback processes.

Our findings pertain to collective behavior studies that emphasize the importance of consistent individual differences or “animal personalities” for the functioning of animal groups. It is well documented that individuals in animal groups behave differently from one another^35–38^, and it is becoming increasingly evident that this heterogeneity plays an important role in shaping collective behaviors^39–47^. It often remains unclear, however, whether the variation we see is the result of underlying differences between individuals (e.g. genetics, physiological state, experience, “personalities”) or actually results from within-group feedback or other stochastic processes^48–55^. Bees in our study showed inter-individual variation in all measured parameters of their response when tested alone, supporting earlier studies on individual variation in bumblebee thermal behavior. While some workers never responded^56^, others did so intermittently, and others in nearly every test trial. Of the responders, some responded with low response thresholds, others with consistently high thresholds. Workers also differed in how much time they invested into fanning. All of these differences in behavior could theoretically contribute to and modulate group responses, but, surprisingly, the variation as shown in isolation did not predict behavior in social context.

For social insects, a large body of literature has focused on the importance of inter-individual variation^51,52^, and differences in response thresholds for task-relevant stimuli are seen as key component for the emergence of division of labor (‘response threshold models’^11,13,23^). In contrast to the prediction of these models, our data show that response thresholds in one context did not predict response behavior in any other context. The combined effect of response probability and response duration when tested alone was most predictive of whether or not an individual would fan in the social contexts. However, the predictive value of this combined effect is so low that its biological relevance is questionable. Also, from one social setting (random social context) to the next (ranked social context), none of the individual parameters predicted response behavior, and bees comprising the subset of fanning individuals changed from trial to trial.

The degree to which a system relies on social responsiveness^56^ (“susceptibility”^57^; “competence”^58^) and individual flexibility presumably depends on the life history of the species, the nature of the task at hand, the degree of collective flexibility required, and the size of the group^6,59,60^, etc. To obtain the collective pattern we found, bumblebees must take into account their social surroundings and optimize their behavior to respond adaptively to the same stimulus. In comparison, in honeybee thermoregulation, genetically determined diversity in response thresholds has been shown to promote the collective maintenance of optimal brood temperature^29,61^. Honeybee colonies are large with up to 50,000 workers, show strong age-based division of labor, and queens are multiple-mated. Bumblebee colonies are small with up to several hundred workers, have weak division of labor^62^, and queens are singly mated. While honeybee colonies can rely on genetic diversity and bet-hedging for variation in worker response^63^, we suggest that bumblebee colonies obtain the collective response pattern by largely overriding individual response tendencies.

Overall, our results highlight the need to quantify individual responses both in respect to the stimulus-response alone and in the actual social context in which they occur in order to assess the possible functional significance of behavioral heterogeneity. By doing this in our experiment, we show that bumblebee colonies use behavioral flexibility to maintain behavioral heterogeneity in task allocation for nest temperature regulation. While individual behavior is flexible and unpredictable, the group response is consistent. We suggest socially mediated behavioral flexibility likely plays an important and potentially overlooked role in many collective phenomena across biological systems.

## Methods

### Bees

Young colonies of *Bombus terrestris* were purchased from a commercial breeder (Biobest, Belgium), reduced to 40–50 workers, and transferred to a wooden box divided into a feeding compartment and a nesting compartment. Colonies were kept in a climate chamber at 24 °C and a 12:12 photoperiod and were provided with sugar solution (Apiinvert, Biobest) and pollen daily.

### Focal individuals

The experiment was conducted in two parts. The first replicate (Rep 1) took place from November to December 2016 and the second replicate (Rep 2) took place in May 2017. For each replicate, we established a foster colony to host individuals collected from multiple origin colonies. All workers in a host colony were individually marked with numbered plastic tags (Opalithplättchen). We then added 90 newly emerged workers (identified by pale coloration that lasts for approximately 24 hours) over the course of three days from 8 colonies for Rep 1 and from 7 for Rep 2, each marked for individual identification (BEETag, code + number;^64^). Experiments began a minimum of 72 hours after the last bee was transferred.

### Experimental setup

Circular test arenas (14 cm diameter, 5.2 cm height) were constructed of 4 mm clear acrylic glass with white plastic floors. Arena walls had 10 holes along the upper edge for ventilation and four holes along the bottom to provide sugar solution and additional ventilation. Each test arena contained eight temperature-controlled brood dummies, pupae-shaped aluminum pins screwed into a water-filled aluminum heating plate and protruding 1 cm into the test arena. The heating plate was attached to a programmable water bath (F25-ME JULABO, USA). Brood dummies protruded through a 2-cm layer of styrofoam separating the metal plate from the arena floor. A red LED light strip was secured around the outside of the test arena wall to illuminate the arena for observation and a glass sheet was placed atop the arena wall to prevent bees from escaping.

### Pre-experiment exposure

To ensure that all workers had experience with temperature stress and the task of fanning in the natural context of the colony before the experiment, the entire foster colony was transferred to a temperature controlled climate chamber and exposed to a change in ambient temperature from 24 °C to 35 °C over the course of 20 minutes and constant 35 °C for 10 minutes. Foster colonies were exposed once before testing started and once between each context.

### Experimental procedure

Experiments were conducted in the climate chamber that housed the colonies. All experiments were performed under red light to maintain the naturally dark in-nest conditions. As bees are insensitive to red light, we are able to observe their thermal behavior while excluding visual interactions and stimuli for the bees during experiments.

Before each trial, brood dummies were covered with a thin layer of parafilm and with 0.025 g of freshly defrosted brood wax (collected earlier from larvae clumps in all origin colonies). Brood dummy temperature was set to 32 °C. Workers were gently removed from the colony with forceps, transferred directly to a test arena and given 10 minutes to habituate with sugar solution provided ad libitum. After the acclimation period, the programmable water bath increased brood-dummy temperature 0.6 degrees/minute from 32° to 46° for 30 minutes. During this time, we continuously observed the bees and recorded whether or not a bee was fanning. Fanning was defined as a worker standing still and fanning her wings for five or more consecutive seconds. Fanning bouts shorter that five seconds were recorded as short fanning. We recorded observations in one-minute bins into notebooks and later transferred the data into Excel. At the end of an experimental trial, workers were returned to their foster colony. Before the next workers were collected, dummy temperature was returned to 32 °C, the arena floor was cleaned with water, and the wax was replaced.

Each individual was tested no more than once a day. Individuals were tested three times on consecutive days in each of the contexts, resulting in 12 trials per individual (but see specifics of sample sizes). Between the different contexts, bees were left undisturbed. The entire experiment took place over the course of 21 days.

### Non-social context

Tagged workers were randomly collected and each transferred to their own arena for testing. 10 bees were tested and observed in parallel. Trials continued until 80 workers had been tested. These bees formed our study subset (per replicate). The procedure was repeated on three consecutive days in random order.

### Random social context

The 80 bees tested in the non-social context were randomly assigned to 10 groups. Groups were assembled into a test arena and tested as in the non-social context. Two groups were tested and observed in parallel. Groups (consisting of the same 10 workers each trial) were reassembled and tested in random order across three consecutive days.

### Ranked social context

Each worker was given a rank based on fanning duration in the random social context. Individuals that that did not fan in the random social context were ranked based on short fanning duration in the random social context, then fanning duration in the non-social context, then short fanning duration in the non-social context. Individuals that showed no fanning across the six previous trials were assigned an equal rank in the last position. The top 10 ranked individuals per replicate were assigned to Rank 1, the next 10 to Rank 2, etc. Resulting ranked social groups were then tested with as in the random social context on three consecutive days in random order.

### Non-social context II

Workers were tested again without nest mates present as in the non-social context.

### Sample size

In Rep 1, bees that never showed fanning in either the non-social context or the random social context were not tested in the ranked social context or the non-social context II; in Rep 2 all bees were tested 12 times across the non-social context, random social context, ranked social context, and non-social context II, unless they died. Overall, four bees died over the course of our experiment (one prior to trials in the random social context; one between trials of the random social context, one prior to the ranked social context, one prior to non-social context II). Our resulting sample sizes are non-social context: 159, random social context: 158, ranked social context: 78 and non-social context II: 77 bees. For analysis of response probability, we excluded bees from Rep. 1 for the ranked social context and non-social context II.

### Stimulus intensity & task availability

To verify that the stimulus environment was comparable across contexts, we measured air temperature in the test arenas, task availability and brood dummy temperature in a control experiment, repeating procedures from the non-social context for 10 bees and from the random social context for 10 groups, using bees from two separate colonies. Five bees and five groups of 10 bees were tested from each colony. Air temperature (ALMEMO 2290–8 data logger) and the number of available brood dummies was recorded per minute. Brood dummies temperature was logged at 1 Hz using temperature sensors (Omega thermocouples) running along the longitudinal axis of the dummies, ending at the tip directly under the wax layer and connected to a data logging device and custom programs (NI cRIO-9074 and NI 9213, LabVIEW 2010 Version 10.0.1, National Instruments, Germany). For statistical analysis, fanning threshold temperature was assigned to the mean wax temperature during the one-minute bin in which long fanning was first observed, as extracted from this data set.

### Statistical Analysis

Data analysis was performed in R 3.2.0^65^. We assess response probability (proportion of individuals that show a fanning response), response threshold (the lowest temperature at which an individual first starts fanning), and response duration (the number of minutes in which an individual shows fanning behavior) within and across contexts. Fanning threshold temperature was assigned to the mean wax temperature during the one-minute bin in which long fanning was first observed. Mean wax temperature per minute was extracted from the sensory environment control experiment data with the stat_smooth function in the R package ggplot2^66^.

To assess inter-individual differences in behavior, we used linear mixed models with threshold and duration as response variables and bee ID as a random factor. To assess individual consistency in behavior we used the R package rptR^67,68^ which used the linear mixed model with trials as a fixed effect and 1000 bootstrap permutations to calculate 95% confidence intervals.

To assess response probability across contexts, we used a logit-link generalized linear mixed model with a binomial error distribution with context as the predictor variable and individual bee as a random effect. To assess response threshold and response duration across contexts, we used a linear mixed model with a Gaussian error distribution. For group-level responses, we used context by random group ID interaction as the fixed effect.

To assess the predictability of response, we used a logit-link generalized linear model with a binomial error distribution with binary response in one context as the response variable and mean variables per bee (and all interactions) from the previous context. We used built-in R function step to obtain a minimum adequate model by AIC comparison. Statistics for non-significant terms were obtained by adding the term to the minimal model. We used the R package rsq^69,70^ to produce likelihood ratio based partial R-squared values for each response variable in the model.

To assess dummy and air temperature consistency, we used a general additive model with temperature as the response variable and context and time as the predictor variables. We used R package itsadug^71^ to calculate the average difference in temperature across time between contexts. To assess dummy availability, we used a linear mixed model with number of available dummies as the response variable and trial type as the predictor variable.

For all models, we calculated p-values from model coefficients, estimated least-square means and 95% confidence intervals, and applied a Tukey post hoc test to calculate pairwise differences between contexts. For GLMM/GLM and LMM/LM analyses, we used the R package lme4^72^. For GAM analyses, we used R package mgcv^73^. For least-square means, 95% confidence intervals, and post hoc tests, we used the R package lsmeans^74^.

## Electronic supplementary material

Supplementary Information

## Data Availability

The datasets generated and analyzed during this study are available in the Open Science Framework repository (https://osf.io/b72td/).
