# Supplementary material for: Behavioral flexibility promotes collective consistency in a social insect
Source: Sci Rep. 2018 Oct 26;8:15836. doi: 10.1038/s41598-018-33917-7 (PMC6203754; doi:10.1038/s41598-018-33917-7)
Supplement: Supplementary file 1 — Supplementary Information [file 41598_2018_33917_MOESM1_ESM.pdf]

Supplementary Information

Behavioral flexibility promotes collective consistency in a social insect

Linda Karen Garrison [1], Christoph Johannes Kleineidam [1], Anja Weidenmüller [1,*]

TABLE S1. Fanning response parameters by treatment and pairwise comparisons between treatments of the 57 bees that showed fanning behavior in both the non-social context and random the social context

	Probability (95% CI)	Threshold (95% CI)	Duration (95% CI)
Non-social	0.85 (0.77 – 9.90)	40.65 (40.18 – 41.11)	8.87 (7.98 – 9.76)
Random social	0.57 (0.48 – 0.66)	42.52 (41.98 – 43.05)	5.53 (4.51 – 6.56)
Ranked social	0.26 (0.19 – 0.35)	43.09 (42.36 – 43.82)	4.40 (3.00 – 5.79)
Non-social II	0.66 (0.57 – 0.74)	41.38 (40.87 – 41.90)	8.15 (7.16 – 9.13)

	Probability<br>z-ratio, p-value	Threshold<br>t-ratio, p-value	Duration<br>t-ratio, p-value
Non-social – Random social	5.334, <.0001	-5.975, <.0001	5.665, <.0001
Non-social – Ranked social	9.563, <.0001	-6.077, <.0001	5.904, <.0001
Non-social – Non-social II	3.792, 0.0009	-2.422, 0.0747	1.268, 0.5839
Random social – Ranked social	5.347, <.0001	-1.359, 0.5263	1.428, 0.4825
Random social – Non-social II	-1.691, 0.3284	3.407, 0.0041	-4.165, 0.0002
Ranked social – Non-social II	-6.777, <.0001	4.127, 0.0003	-4.807, <.0001

TABLE S2. Fanning response parameters by treatment and pairwise comparisons between treatments of the 32 bees that showed fanning behavior in all four contexts.

	Probability (95% CI)	Threshold (95% CI)	Duration (95% CI)
Non-social	0.84 (0.75 – 0.91)	40.84 (40.12 – 41.56)	8.91 (7.56 – 10.25)
Random social	0.60 (0.49 – 0.70)	42.17 (41.38 – 42.97)	6.48 (4.99 – 7.97)
Ranked social	0.48 (0.37 – 0.59)	43.07 (42.23 – 43.92)	4.74 (3.14 – 6.34)
Non-social II	0.80 (0.70 – 0.87)	41.61 (40.88 – 42.34)	8.46 (7.10 – 9.83)

	Probability<br>z-ratio, p-value	Threshold<br>t-ratio, p-value	Duration<br>t-ratio, p-value
Non-social – Random social	3.639, 0.0016	-3.574, 0.0024	3.309, 0.0059
Non-social – Ranked social	5.017, <.0001	-5.595, <.0001	5.309, <.0001
Non-social – Non-social II	0.751, 0.8763	-2.248, 0.1136	0.657, 0.9128
Random social - Ranked social	1.626, 0.3640	-2.113, 0.1521	2.076, 0.1642
Random social - Non-social II	-2.986, 0.0150	1.486, 0.4477	-2.672, 0.0401
Ranked social – Non-social II	-4.442, 0.0001	3.636, 0.0019	-4.710, <.0001

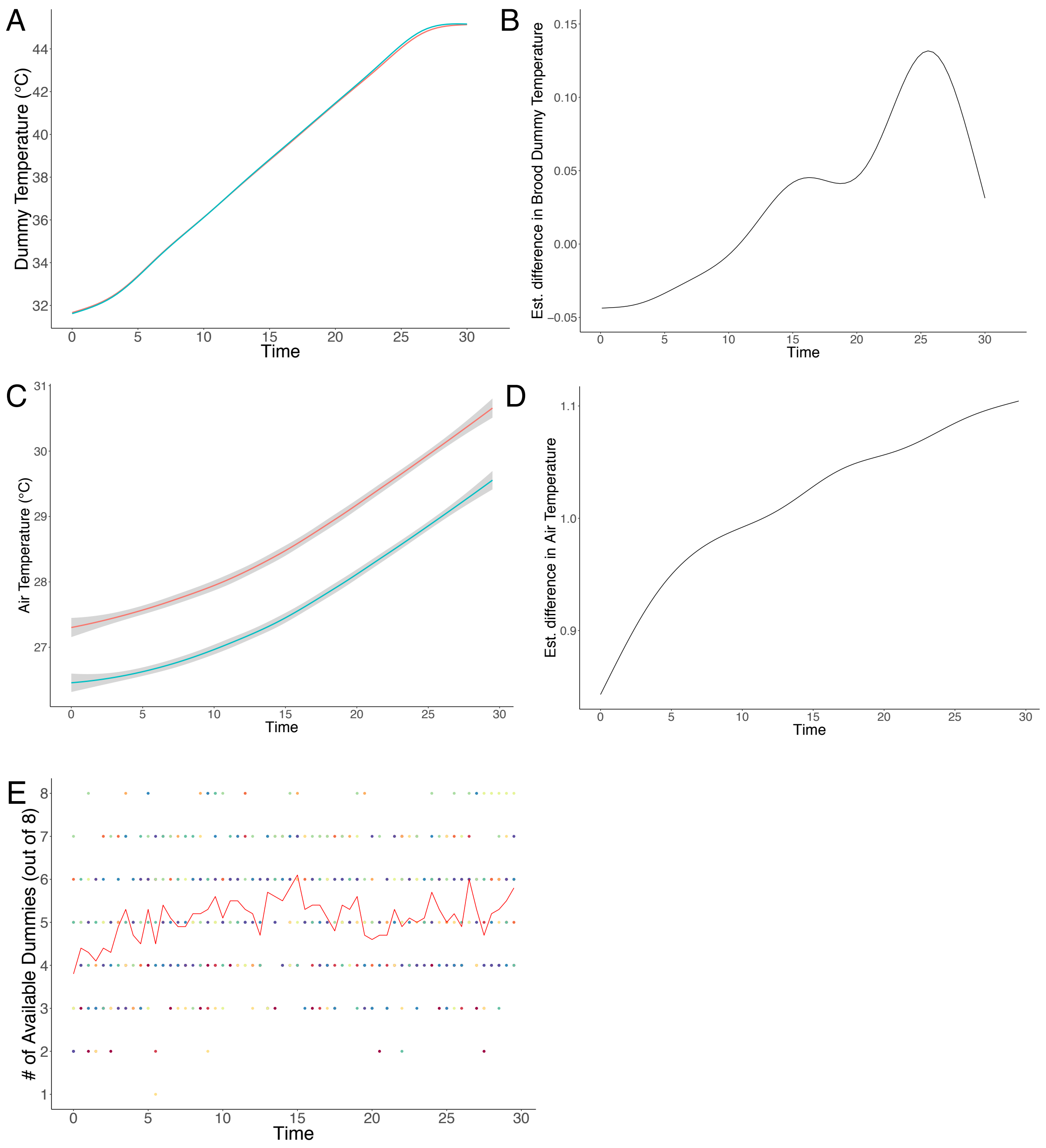

Figure S1. Stimulus intensity & task availability.

(A) Mean brood dummy temperature across time. Blue denotes the 10 trials with single individuals; orange denotes the 10 group trials. (B) The average difference in mean brood dummy temperature across time between contexts. Maximum difference: 0.132°C. (C) Test arena air temperature across time. (D) Maximum difference: 1.10°C (E) The number of brood dummies available across time in group trials. Average dummy availability: 5.11 [5.03 - 5.18].

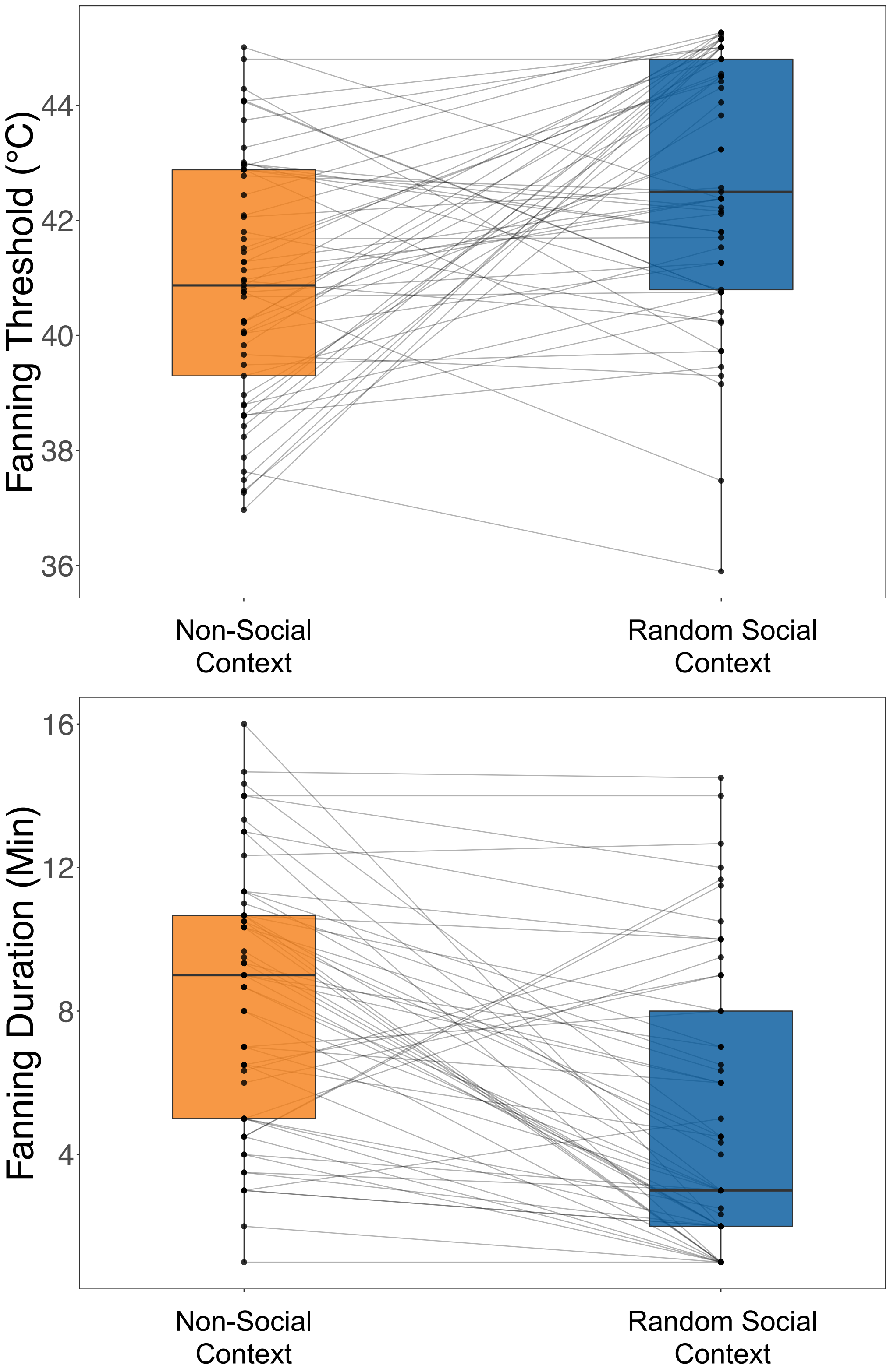

Figure S2. The 57 individuals that responded in both the non-social context and the random social context, fanning response parameters by context. (A) Boxplots depicting mean fanning thresholds and (B) mean fanning durations for each context. Each dot depicts one individual (n=57).

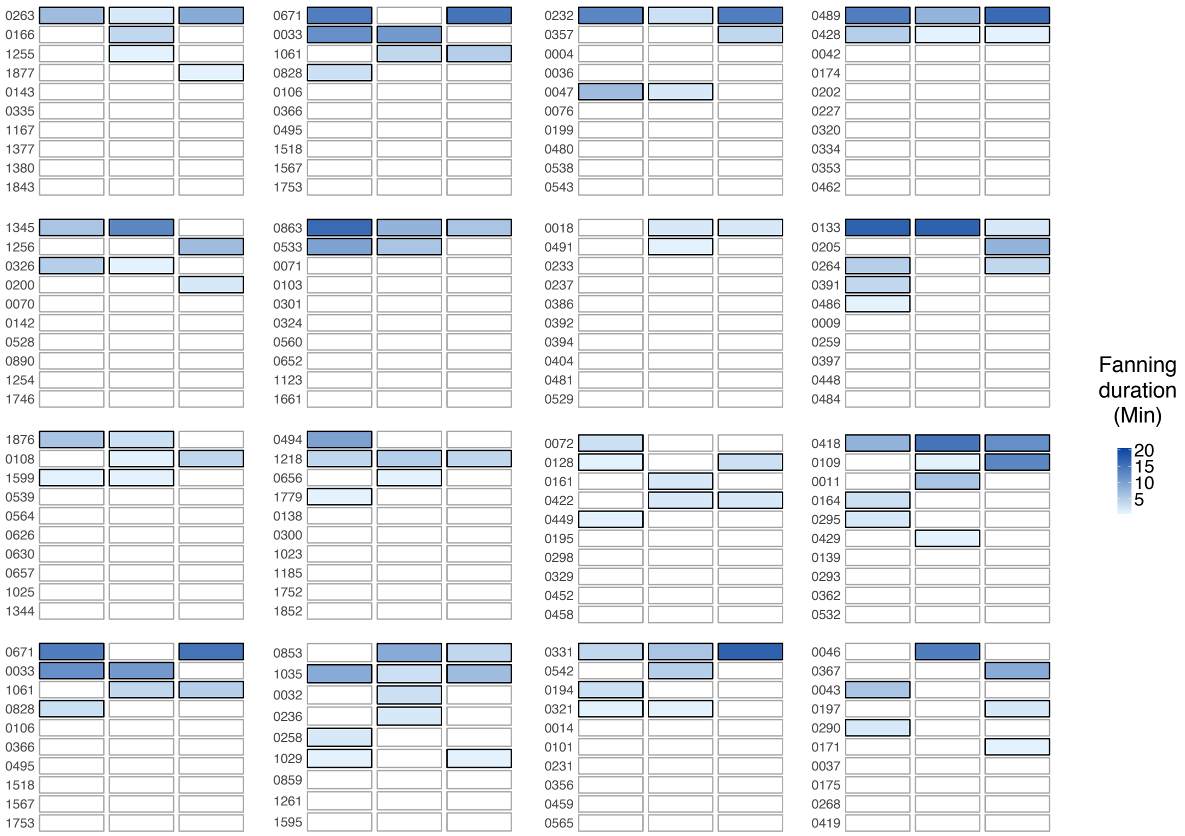

Figure S3. Individual fanning response in the random social context, all groups. Individuals (rows) were randomly assembled into 16 groups of ten and tested three times (columns). Gradient indicates fanning duration per trial; open boxes indicate no fanning.

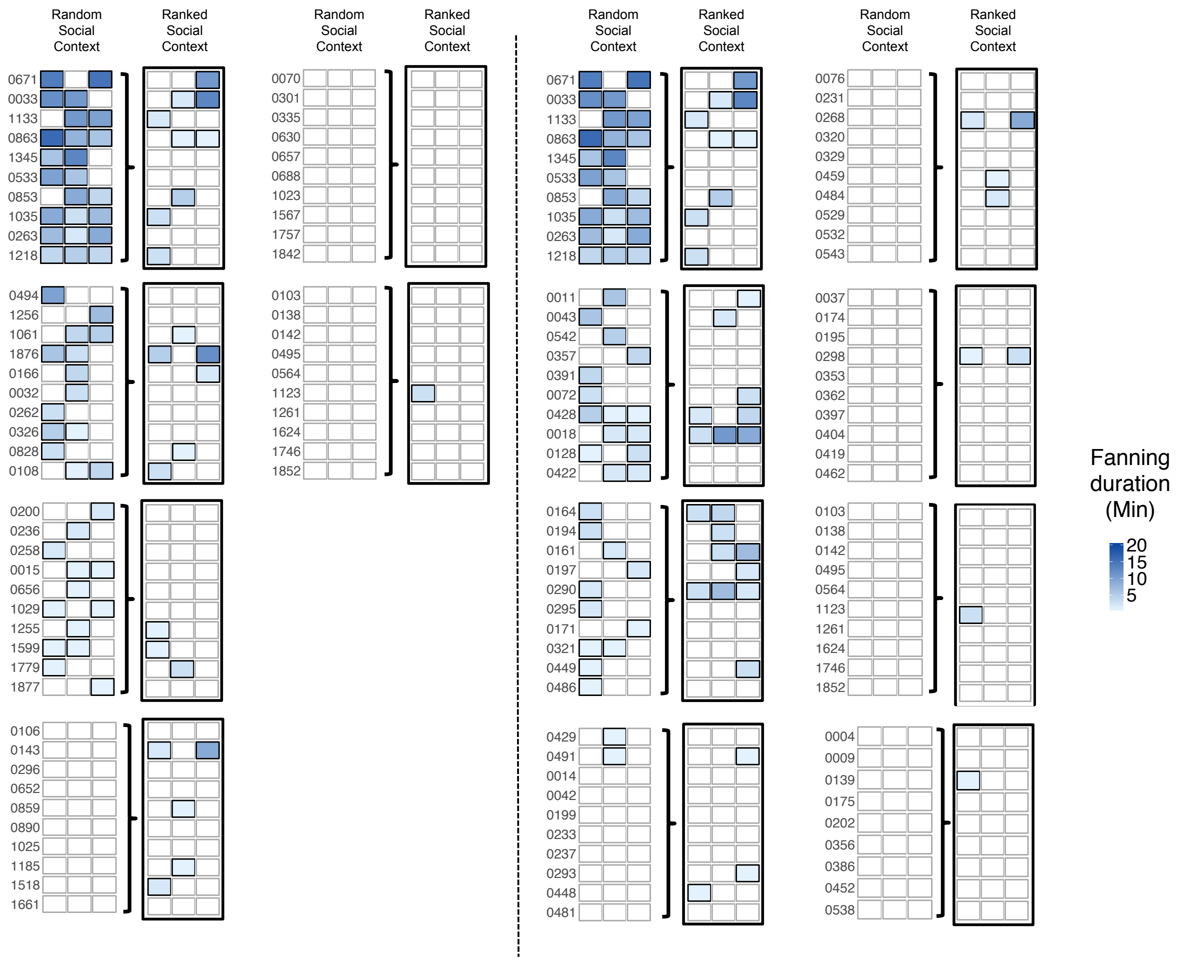

Figure S4. Individual fanning response, random social to ranked social, all groups
(Left) Fanning response behavior of individuals (rows) across three trials (columns) of the random social context. Individuals are sorted by mean fanning duration in the random social context. Fanning duration per trial is indicated by gradient, open boxes indicate no fanning. (Right) Fanning response behavior of same individuals across three trials of ranked social context. Individuals were assembled into groups of ten (black box) based on similarity in their fanning behavior in the random social context. The eight groups on the left are from Rep 1 and the eight groups on the right are from Rep 2.

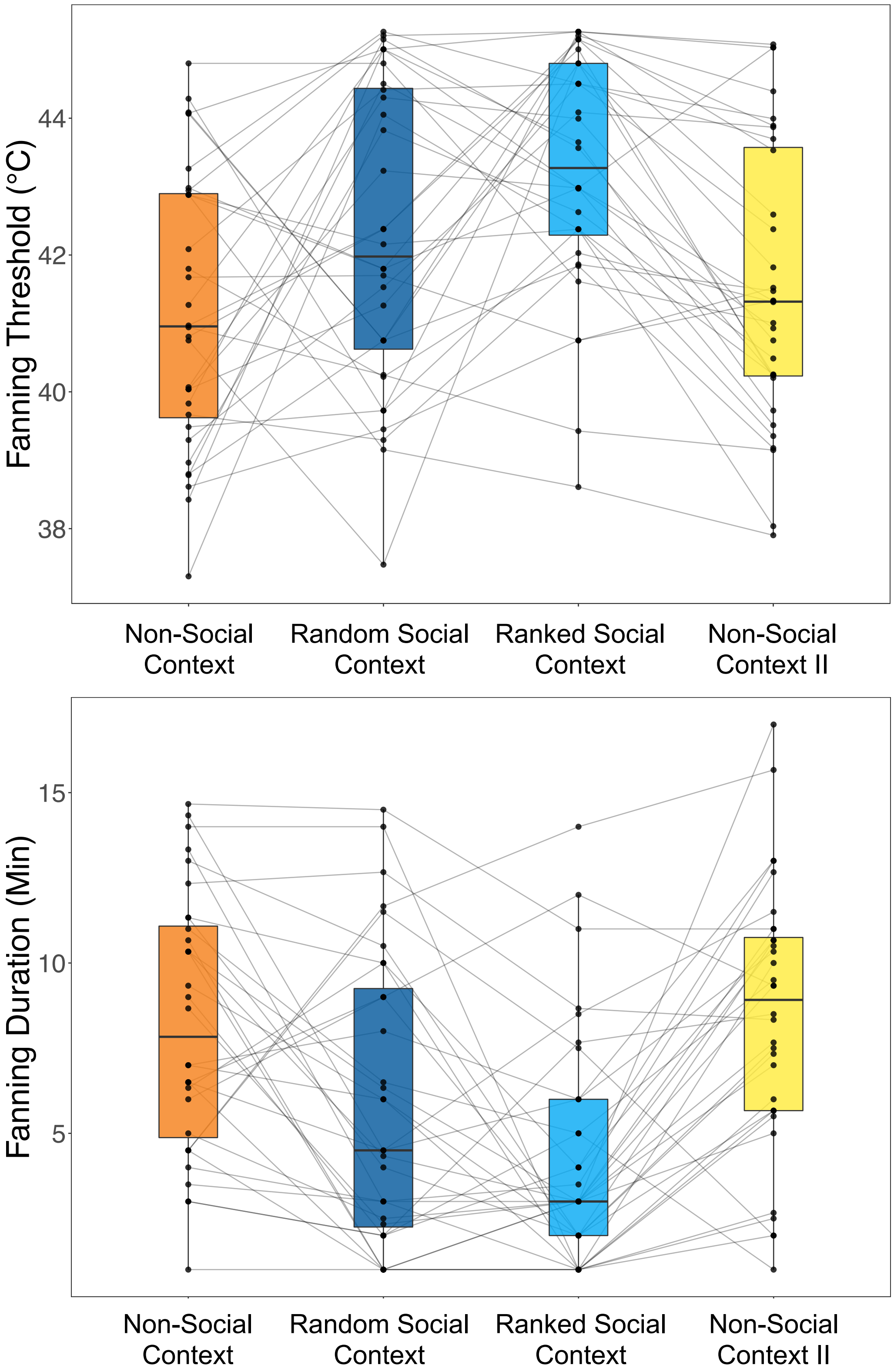

Figure S5. The 32 individuals that responded in all four contexts, fanning response parameters by context. (A) Boxplots depicting mean fanning thresholds and (B) mean fanning durations for each context. Each dot depicts one individual (n=32).
